# The sVEGFR1-i13 splice variant regulates a β1 integrin/VEGFR autocrine loop involved in the progression and the response to anti-angiogenic therapies of squamous cell lung carcinoma

**DOI:** 10.1038/s41416-018-0128-4

**Published:** 2018-05-24

**Authors:** Cherine Abou Faycal, Elisabeth Brambilla, Jackeline Agorreta, Nina Lepeltier, Thibault Jacquet, Nicolas Lemaître, Anouk Emadali, Anthony Lucas, Pedro M Lacal, Luis Montuenga, Ruben Pio, Sylvie Gazzeri, Beatrice Eymin

**Affiliations:** 1 Team RNA Splicing, Cell Signalling and Response to Therapies, Institute For Advanced Biosciences, INSERM U1209, CNRS UMR5309, Université Grenoble Alpes, 38000 Grenoble, France; 2 Team Tumor Molecular Pathology and Biomarkers, Institute For Advanced Biosciences, INSERM U1209, CNRS UMR5309, Université Grenoble Alpes, 38000 Grenoble, France; 3 Program in Solid Tumors (CIMA), CIBERONC and Navarra Institute for Health Research (IdiSNA), 31008 Pamplona, Spain; 4 Department of Histology and Pathology, University of Navarra, School of Medicine, 31008 Pamplona, Spain; 5 Team Genetics and Epigenetics of Lymphoid Cancers, Institute For Advanced Biosciences, INSERM U1209, CNRS UMR5309, Université Grenoble Alpes, 38000 Grenoble, France; 6 Laboratory of Molecular Oncology, Istituto Dermopatico dell’Immacolata IRCCS, Rome, Italy; 7 Department of Biochemistry and Genetics, University of Navarra, School of Sciences, 31008 Pamplona, Spain

**Keywords:** Non-small-cell lung cancer, Targeted therapies

## Abstract

**Background:**

While lung adenocarcinoma patients can somewhat benefit from anti-angiogenic therapies, patients with squamous cell lung carcinoma (SQLC) cannot. The reasons for this discrepancy remain largely unknown. Soluble VEGF receptor-1, namely sVEGFR1-i13, is a truncated splice variant of the cell membrane-spanning VEGFR1 that has no transmembrane or tyrosine kinase domain. sVEGFR1-i13 is mainly viewed as an anti-angiogenic factor which counteracts VEGF-A/VEGFR signalling in endothelial cells. However, its role in tumour cells is poorly known.

**Methods:**

mRNA and protein status were analysed by Real-Time qPCR, western blotting, ELISA assay, proximity ligation assay or immunohistochemistry in human tumour cell lines, murine tumourgrafts and non small cell lung carcinoma patients samples.

**Results:**

We show that anti-angiogenic therapies specifically increase the levels of sVEGFR1-i13 in SQLC cell lines and chemically induced SQLC murine tumourgrafts. At the molecular level, we characterise a sVEGFR1-i13/β1 integrin/VEGFR autocrine loop which determines whether SQLC cells proliferate or go into apoptosis, in response to anti-angiogenic therapies. Furthermore, we show that high levels of both sVEGFR1-i13 and β1 integrin mRNAs and proteins are associated with advanced stages in SQLC patients and with a poor clinical outcome in patients with early stage SQLC.

**Conclusions:**

Overall, these results reveal an unexpected pro-tumoural function of sVEGFR1-i13 in SQLC tumour cells, which contributes to their progression and escape from anti-angiogenic therapies. These data might help to understand why some SQLC patients do not respond to anti-angiogenic therapies.

## Introduction

Lung cancer is the most commonly diagnosed cancer. It also has the highest mortality rate among all cancers. Over 85% of lung cancers are classified as non-small cell lung cancer (NSCLC). NSCLCs are comprised of adenocarcinoma (ADC) and squamous cell carcinoma (SQLC) that make up ~50 and 30% of lung cancers respectively.^1^ In pre-clinical mouse models, we previously demonstrated that treatment with DC101, a murine anti-VEGFR2 antibody, or sunitinib, a VEGFR-TKI, promotes tumour growth in SQLC but not in lung ADC.^2^ In addition, clinical trials have shown that SQLC patients exhibit severe complications with sorafenib a VEGFR tyrosine kinase inhibitor, or fatal haemorragies upon treatment with bevacizumab, a humanised anti-VEGF-A monoclonal antibody, restricting the administration of these treatments to non squamous patients.^3,4^ Therefore, the efficacy and safety of anti-angiogenic therapies in NSCLC appear to be closely dependent of the histological sub-type. To date, the molecular bases of this differential response between both histological subtypes are unknown and there are no validated biomarkers to select SQLC patients eligible for these therapies.

Vascular endothelial growth factor receptor-1 (VEGFR1) is a tyrosine kinase receptor for members of the vascular endothelial growth factor (VEGF) family. In addition to the transmembrane isoform of VEGFR1, different cell types, including endothelial and tumour cells, produce extra-cellular forms of VEGFR1 that are devoid of VEGFR1 transmembrane and tyrosine kinase domains. They are generally referred as sVEGFR1. sVEGFR1 may come from proteolytic cleavage and ectodomain shedding of membrane VEGFR1, as well as from *VEGFR1* pre-mRNA alternative splicing.^5^ To date, four alternatively spliced *VEGFR1* transcripts have been described, namely *sVEGFR1-i13*, *sVEGFR1-i14*, *sVEGFR1-e15a* and *sVEGFR1-e15b*. They are all common through to exon 13 but differ in their unique C-terminus (Supplementary Figure 1A).^6^ Among these splice variants, *sVEGFR1-i13* appears to be the most abundant isoform in many tissues. At the functional level, it is a widely held view that circulating truncated sVEGFR1s negatively regulate endothelial cells proliferation and inhibit angiogenesis by sequestering VEGF-A or by acting as dominant negative via heterodimerisation with membrane-spanning VEGFR1 and VEGFR2.^7^ Consistently, sVEGFR1 inhibits tumour neovascularisation, growth and metastasis in several mouse tumour models,^8,9^ and low expression of sVEGFR1 is associated with enhanced angiogenesis and a poor prognosis in breast cancer patients.^10^ On the basis of its anti-angiogenic functions, it has also been proposed that plasmatic sVEGFR1 serves as a predictive biomarker of response to anti-angiogenic therapies, notably to bevacizumab.^11^ As an example, high levels of circulating sVEGFR1 pre- or post-bevacizumab treatment correlated with worse survival in patients with triple negative breast cancers or NSCLCs, respectively.^12,13^ This poor response was associated with insufficient baseline microvascular density. However, other studies have complicated this simple view. Hence, sVEGFR1 was found to promote adhesion and migration of endothelial cells through interaction with α5β1 integrin and activation of VEGFR2 signalling, thus acting rather as a pro-angiogenic molecule.^14,15^ In addition, sVEGFR1 was reported to trigger non-apoptotic cell death in ovarian and colorectal cancer cell lines, indicating that sVEGFR1 could also inhibit tumour growth directly.^16^ However, the characterisation, if they exist, of specific functions associated with sVEGFR1 splice variants remain poorly understood in cancer cells, notably during the response to therapies. Less is even known about the molecular mechanisms and upstream signals that contribute to the generation of sVEGFR1 in cancer cells. In this study, we demonstrate that a cross-talk between sVEGFR1-i13, β1 integrin and VEGFR is specifically involved in the progression and the response of SQLC cells to various anti-angiogenic therapies.

## Materials and methods

### Cells, cell culture and reagents

Cells were cultured as described previously.^17^ Bevacizumab (Avastin®) was provided by Roche/Genentech. VEGFR2 kinase inhibitor KI8751 was from Calbiochem and VEGFR1/R2 kinase inhibitor SU5416 from Sigma-Aldrich. The rhVEGFR1/Flt-1 Fc Chimera was obtained from R&D Systems. pBLAS-Control and pBLAS49.2/sVEGFR1 encoding sVEGFR1-i13 were previously described.^18^ Transfections of plasmid DNA were performed using X-tremeGENE 9 (Roche). Cells were analysed 48 h after transfection. The peptides p12 (NYLTHRQ) and sp12 (LTQNYRH) were from Covalab. They were resuspended in water at 10 mg/ml and used at a final concentration of 10 µg/ml.

### Patients and tissue samples

Seventy-seven human NSCLC and 17 matched normal lung parenchymas were included in this study. Tumours consisted of 41 ADC and 36 SQLC. Tumour tissues and normal lung parenchyma, taken away from the bulk of the tumour, were collected from resection of lung tumours, and stored for scientific research in a biological resource repository (Centre de Ressources Biologiques, CHU Albert Michallon, Grenoble Hospital) following national ethical guidelines. All patients enrolled in this trial provided written informed consent. Tissue banking and research conduct was approved by the Ministry of Research (approval AC-2010-1129) and by the regional IRB (CPP 5 Sud Est). For histological classification, tumour samples were fixed in formalin, and diagnosis was made on paraffin-embedded material using the WHO VII^th^ classification of lung criteria.^19^ For each case, one section from the most representative block was chosen. These sections always contained more than 70% tumour cells.

### RNA interference and morpholinos

The two siRNAs specifically targeting sVEGFR1-i13 were: sVEGFR1-i13(1) sense, 5′-UAACAGUUGUCUCAUAUC-3′; anti-sense, 5′-UGAUAUGAGACAACUGUUA-3′ and sVEGFR1-i13(2) sense, 5′-UCUCGGAUCUCCAAAUUU-3′; anti-sense, 5′-UAAAUUUGGAGAUCCGAGA-3′. Transfection of siRNA oligonucleotide duplexes was performed using JetPrime reagent (Ozyme). β1 integrin silencing was performed using SMARTpool siGENOME ITGB1 siRNA (M-004506-00-0020, Thermoscientific Dharmacon). Transfection of smartpools was performed using Oligofectamine RNAiMax (Invitrogen). The scrambled siRNA oligonucleotides used as a control for all RNA interference experiments were as follows: forward 5′-UCGGCUCUUACGCAUUCAATT-3′ and reverse 5′-CAAGAAAGGCCAGUCCAAGTT-3′. Cells were analysed 72 h post-transfection. Morpholino oligos were obtained from Gene Tools and resuspended in sterile water as a 200 µM stock solution. Sequences of control and sVEGFR1-i13 (MoFL2) morpholino oligos were as follows: 5′- GATCCATCCCTCTGTTAAGACCTAG-3′ and 5′- TTTTTGTTGCAGTGCTCACCTCTGA-3′, respectively. For delivering, MGH7 or H2170, cells were seeded at 0.5 × 10^6^ cells/well in six-well plates for 24 h, treated with 10 µM of control or MoFL2 morpholino, then scraped using a rubber blade scraper and transfered to another culture plate. Cells were analysed at 24, 48 and 72 h after transfection.

### Apoptosis, cell proliferation, clonogenic, ELISA and soft agar assays

Apoptosis was evaluated by scoring the percentage of apoptotic nuclei in 200 cells after staining with Hoescht-33258 (Calbiochem). Detection of sVEGFR1 in supernatants or cell pellets was performed by ELISA using the Quantikine sVEGFR1 kit (R&D Systems). Detection of active caspase-3, MTS cellular proliferation, clonogenic and soft agar assays were performed as described previously.^2,20,21^

### RNA extraction, reverse transcription and real-time qPCR analysis

Total RNA was extracted using the High Pure RNA Isolation Kit (Roche Diagnostics) or TRIzol (Thermo Fisher Scientific) according to the manufacturer’s instructions. In total, 1 µg of total RNA was subjected to Reverse Transcription using iScript RT supermix (Bio-Rad). Quantitative RT-PCR (qRT-PCR) was performed using iTaq® qPCR Universal SYBR Green Supermix (Bio-Rad). The primer sequences used were as follows: *sVEGFR1-i13*: 5′-AGGGGAAGAAATCCTCCAGA-3′(forward) and 5′-CAACAAACACAGAGAAGG-3′(reverse); *sVEGFR1-e15a*: 5′-ACACAGTGGCCATCAGCAGTT -3′(forward) and 5′-CCCGGCCATTTGTTATTGTTA-3′(reverse); *sVEGFR1-i14*: 5′-ACAACAAGAGCCTGAACTGTA-3′(forward) and 5′-ATAACAAATGGCCGGGCATGG-3′(reverse); *VEGFR1(1)*: 5′-AGGGGAAGAAATCCTCCAGA-3′ (forward) and 5′-CGTGCTGCTTCCTGGTCC-3′(reverse); *VEGFR1(2):* 5′-ACCGAATGCCACCTCCATG-3′(forward) and 5′-AGGCCTTGGGTTTGCTGTC-3′(reverse); *VEGFR2:* 5′-TCAAAGGAGAAGCAGAGC-3′(forward) and 5′-GCACTCTTCCTCCAACTGCCAATA-3′(reverse); *GAPDH*: 5′-CGAGATCCCTCCAAAATCAA-3′(forward) and 5′-ATCCACAGTCTTCTGGGTGG-3′(reverse). Relative gene expression was calculated, for each sample, as the ratio of specific target gene to GAPDH gene (housekeeping gene), thus normalising the expression of target gene for sample-to-sample differences in RNA input.

### Antibodies and immunoblotting

Immunoblotting experiments were performed as previously described.^21^ The antibodies used were: anti-sVEGFR1-i13 polyclonal antibody, generated against a peptide mapping in the unique C-terminus,^14^ anti-VEGFR2 (clone 55B11), anti-actin and anti-active caspase-3 from Cell signalling. Anti-tubulin was from Santa Cruz, anti-actin from Sigma-Aldrich, anti-phospho-VEGFR1-Tyr1213 from Millipore and anti-phospho-VEGFR2-Tyr1214 from Invitrogen. Antibody against total β1 integrin (serum 227) was kindly provided by Dr Albiges-Rizo (Institute For Advanced Biosciences, Grenoble). The other antibodies used for Proximity Ligation Assay and immunohistochemistry were: mouse anti-β1 integrin (4B7R) and anti-VEGFR2 (4B4) antibodies from ThermoFisher Scientific, rabbit anti-VEGFR2 from Sigma-Aldrich and anti-VEGFR1 (Santa Cruz).

### Immunoprecipitation experiments

MGH7 or H2170 cells treated or not were lysed on ice using RIPA buffer (150 mM NaCl, 50 mM Tris HCl pH 8, 0.1% SDS, 1% Nonidet P40, 0.5% Na deoxycholate) supplemented with proteases and phosphatase inhibitors and 5 mM sodium molybdate (Sigma Aldrich). In total, 1 mg of total protein extracts were pre-cleared for 1 h in the presence of a mixture of protein A-magnetic beads and incubated overnight at 4 °C, together with protein A beads, with an anti-VEGFR2 antibody (55B11). An irrelevant rabbit IgG (PP64, Millipore) was used as control for immunoprecipitation.

### Immunohistochemistry on patients and subcutaneous tumourgraft samples

Immunohistochemical analysis was carried out on formalin-fixed and paraffin-embedded tissue sections as previously described.^20^ For immunostaining evaluation, a score (0–300) was established by multiplying the percentage of labelled cells (0–100%) by the staining intensity (0, null; 1, low; 2, moderate; 3, strong). For sVEGFR1-i13, both nuclear and cytoplasmic scores were considered and added to get a total sVEGFR1-i13 score for each sample. Scores obtained for alveolar type II pneumocytes and bronchial cells in normal lung tissues taken at distance from the tumour were considered as normal scores for ADC and SQLC, respectively. According to median scores in normal tissues and to the histograms of distribution, tumours were sub-divided in two classes for sVEGFR1-i13 (class 0: low ≤ 100; class 1: high > 100) and β1 integrin (class 0: low ≤ 150; class 1: high > 150). Sections from UN-SCC680 and UN-ADC12 subcutaneous tumourgrafts embedded in paraffin were recovered from previous experiments^2^ and stained for sVEGFR1-i13 or P-VEGFR1(Tyr1213). For automatic quantification of sVEGFR1-i13 staining in mouse models, sections were scanned using a ZEISS AxioImager M2 automated slide scanner with ×5 magnification and the images were analysed using Image J software. Threshold values were adjusted until masked brownpixels correlated with positive immunostaining or with total area of the digitised tissue. The percentage of positive areas was then calculated for each staining.

### Proximity ligation assay (PLA)

MGH7 cells were seeded on 8-wells Lab-tek II and treated or not for 24 h with recombinant sVEGFR1 (10 ng/ml) or SU5416 (10 µM) or for 72 h with bevacizumab (10 µg/ml). PLA was performed using the Duolink^R^ In Situ kit from Sigma-Aldrich according to the manufacturer’s recommendations. A multiphoton Zeiss (Oberkochen Germany) LSM510 META NLO confocal microscope was used to analyse immunofluorescence experiments at ×60 magnification. Images were acquired with AxioCam digital microscope camera and analysed using ICY 1.7 software. All images are z-stacked.

### Statistical analyses

The statistical analyses (Student *t*-test or ANOVA) were performed using Statview software (Abacus Concepts). Descriptive analyses comparing continuous and two- or three-level categorical variables were carried out using the Mann–Whitney *U* test or the Kruskal–Wallis test, respectively. The Chi2 test was used to test the association between two categorical variables. Univariate survival analyses were done using the Kaplan–Meier method and *p* values were derived from a log-rank test. Overall survival (OS) was calculated from the date of surgery to the date of death. *p* values < 0.05 were considered significant.

## Results

### Anti-angiogenic therapies induce the accumulation of sVEGFR1-i13 in squamous lung carcinoma

**Fig. 1:**
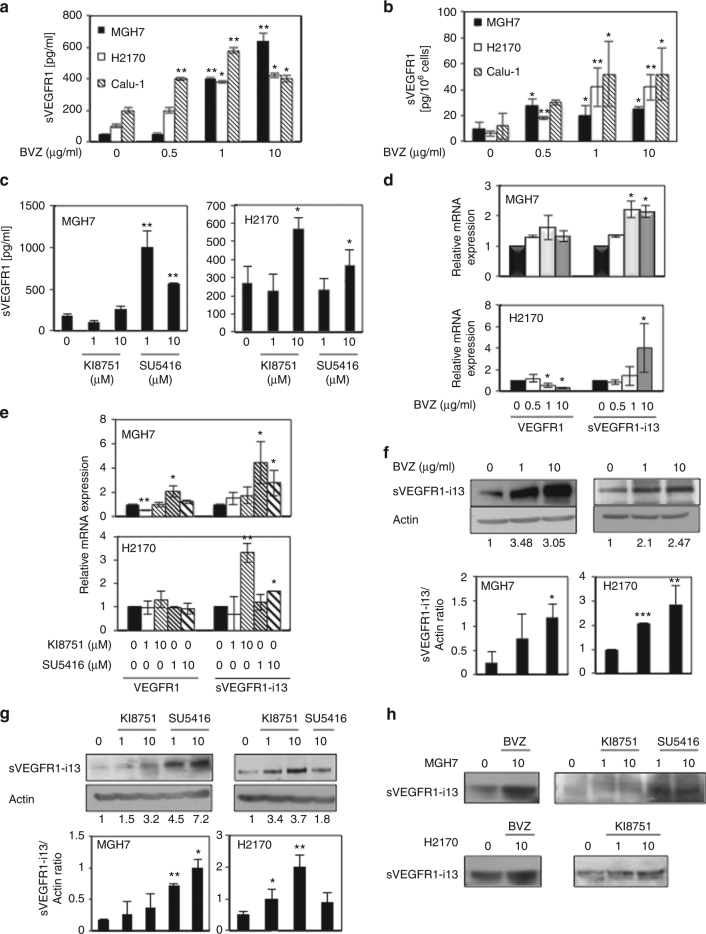
Anti-angiogenic therapies upregulate sVEGFR1-i13 expression in squamous cell lung carcinoma cell lines. The MGH7, H2170 or Calu-1 SQLC cell lines were treated with the indicated concentrations of bevacizumab (µg/ml) for 72 h (**a**, **b**, **d**, **f**, **h**) or with the indicated concentrations (µM) of KI8751 or SU5416 for 24 h (**c**, **e**, **g**, **h**). **a–c** ELISA assays were performed for quantification of sVEGFR1 protein level in the supernatants (**a**, **c**) or the cell pellets (**b**). **d**, **e** RT-qPCR analyses were performed to quantify *sVEGFR1-i13* and *VEGFR1* mRNA levels. *GAPDH* was used as an internal control. The value 1 was arbitrarily assigned to the specific signal/gapdh ratio obtained in non treated cells. **f**, **g** Western-blotting analyses of sVEGFR1-i13. Actin was used as a loading control. Histograms represent the quantification of the specific signal relative to actin. In all experiments, the means ± SD of three independent experiments are shown. (*p < 0.05; **p < 0.01; ***p < 0.001). (**h**) Western-blotting analyses of sVEGFR1-i13 in the supernatants of treated cells. Staining of the PVDF membrane using red ponceau was done to verify equal loading in all samples (not shown)

In pre-clinical studies, we previously demonstrated that squamous lung carcinomas do not respond to anti-angiogenic therapies.^2^ On the basis of its anti-angiogenic functions, it has been proposed that plasmatic sVEGFR1 serves as a predictive biomarker of response to anti-angiogenic therapies, notably to bevacizumab.^11^ Therefore, we tested the impact of anti-angiogenic treatments on sVEGFR1 protein expression in various SQLC cell lines. Using ELISA assays, we showed that extra-cellular (Fig. 1a) and also intra-cellular (Fig. 1b) sVEGFR1 protein levels increase in a dose-dependent manner in response to bevacizumab in MGH7, H2170 and Calu-1 cell lines. Same results were obtained in the supernatants of MGH7 and H2170 cells treated with two VEGFR tyrosine kinase inhibitors, namely KI8751 and SU5416 (semaxanib) (Fig. 1c). As ELISA assays do not distinguish between the different sVEGFR1 isoforms, we analysed whether anti-angiogenic treatments affect sVEGFR1 splice variants. We performed RT-qPCR in MGH7 and H2170 cells using specific primers. We observed a significant increase of *sVEGFR1-i13* mRNA level in response to bevacizumab (Fig. 1d), SU5416 or KI8751 (Fig. 1e). In addition, using an antibody that specifically recognises the sVEGFR1-i13 C-terminus,^14^ we confirmed the accumulation of this protein variant in response to anti-angiogenic treatments, both in cellular protein extracts (Fig. 1f, g) and in the supernatants of treated cells (Fig. 1h). In contrast, *sVEGFR1-e15a* and *sVEGFR1-i14* mRNA levels did not significantly vary in MGH7 cells treated in the same conditions (Supplementary Figure 1B). In H2170 cells, we noticed a decrease of *sVEGFR1-i14* mRNA level in response to SU5416 and bevacizumab and a slight increase of *sVEGFR1-e15a* mRNA level in response to KI8751 (Supplementary Figure 1C). The *sVEGFR1-e15b* transcript was never detected in our cells. Interestingly, when various lung ADC or large cell carcinoma cell lines were treated with anti-angiogenic therapies, secreted sVEGFR1 (Supplementary Figure 2A) or sVEGFR1-i13 protein (Supplementary Figure 2B) and mRNA (Supplementary Figure 2C) levels did not go up, and even decreased.

**Fig. 2:**
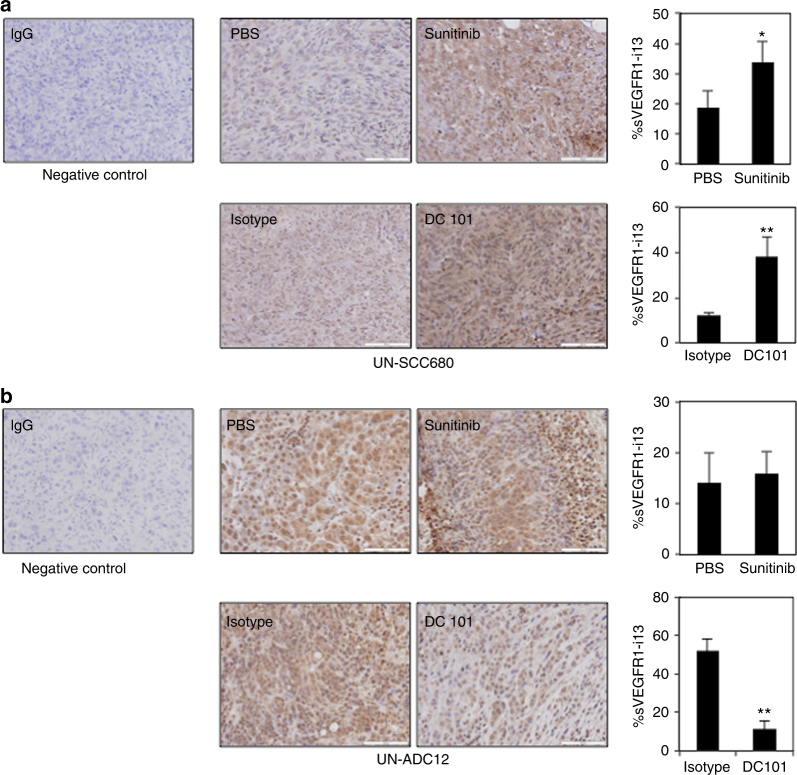
Anti-angiogenic treatments upregulate sVEGFR1-i13 in murine SQLC tumourgrafts. **a**, **b** Left panels: representative immunostainings of paraffin-embedded sections of UN-SCC680 (**a**) or UN-ADC12 (**b**). The tumourgrafts had received PBS, control isotype, sunitinib or DC-101 as indicated. Negative immunostaining (irrelevant IgG, negative control) is also shown. Right panels: automatic quantification of tumour cell immunostaining (as described in the Materials and methods section) for each condition. The mean ± SD of 6 mice per condition is shown. Statistical analyses were performed using a student non paired *t*-test (*p < 0.05; **p < 0.01)

To confirm these results, we took advantage of two mouse tumourgraft models carried out with ADC (UN-ADC12) or SQLC (UN-SCC680) cells derived from chemically-induced tumours and having received or not sunitinib, a VEGFR TKI, or DC101, a murine anti-VEGFR2 antibody.^2^ Consistent with our results in cell lines, increased sVEGFR1-i13 immunostaining was detected in UN-SCC680 tumourgrafts treated with both anti-angiogenic therapies compared to untreated tumourgrafts (Fig. 2a). In contrast, absence of variation or decreased staining of sVEGFR1-i13 was observed in UN-ADC12 tumourgrafts treated with sunitinib or DC101, respectively (Fig. 2b). These results were confirmed in vitro using SCC680 (Supplementary Figure 2D) or ADC12 (Supplementary Figure 2E) murine cells treated with SU5416 or KI8751, respectively. Taken together, these data demonstrate that various anti-angiogenic therapies increase sVEGFR1-i13 expression in squamous cell lung carcinoma but not in lung ADC.

### sVEGFR1-i13 regulates a VEGFR autocrine loop in SQLC tumour cells in response to anti-angiogenic therapies

**Fig. 3:**
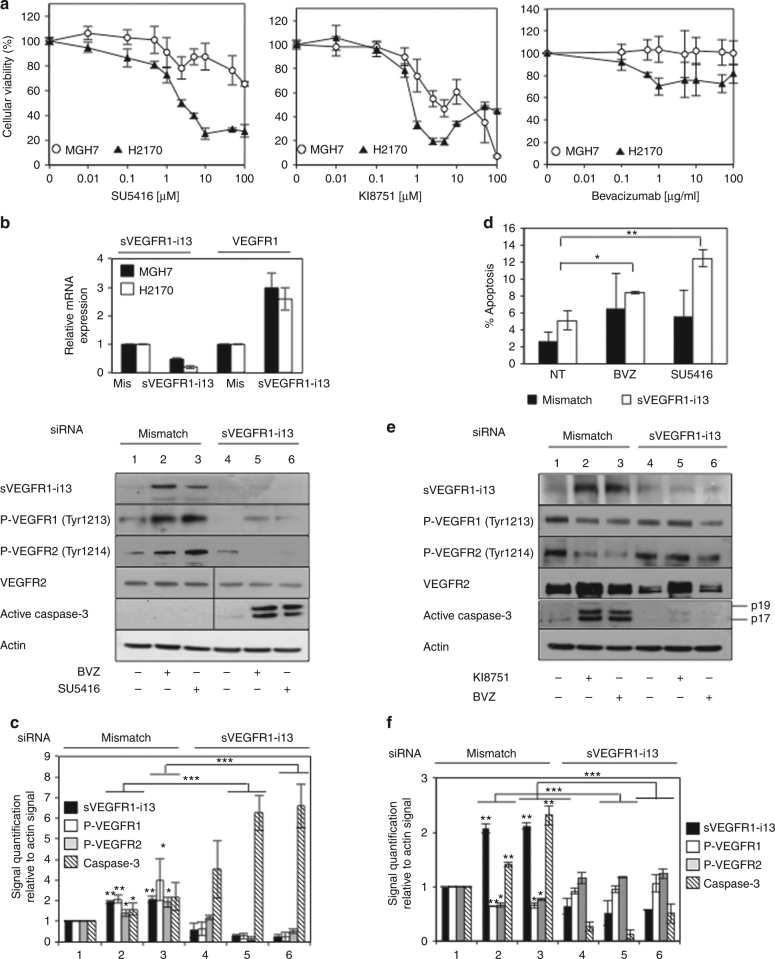
**a** sVEGFR1-i13 regulates in an opposite way VEGFR1/VEGFR2 signalling pathways in response to anti-angiogenic therapies. (**b**) 96 h MTS proliferative assays were performed in MGH7 and H2170 cells treated with increasing concentrations of SU5416, KI8751 or bevacizumab as indicated. The mean ± SD of three independent experiments are shown. **b**–**f** MGH7 or H2170 cells were transfected for 72 h with two distinct *sVEGFR1-i13* siRNA (or *mismatch* siRNA) and treated with 10 µg/ml bevacizumab (BVZ). Alternatively, the cells were transfected for 48 h with *sVEGFR1-i13* siRNA and treated for 24 additional hours with 10 µM SU5416 or 10 µM KI8751. **b** Upper panel: RT-qPCR analyses were performed in MGH7 or H2170 cells to quantify *sVEGFR1-i13* and *VEGFR1* mRNA levels and assess the efficiency of the knock-down of sVEGFR1-i13. *GAPDH* was used as an internal control. The value 1 was arbitrarily assigned to the specific signal/gapdh ratio obtained in mismatch transfected cells. Lower panel: Western blot analyses in MGH7 cells of the indicated proteins are shown. Actin was used as a loading control. **c** Quantification of the signal for each indicated protein relative to actin in three different experiments. sVEGFR1-i13 (black histograms), P-VEGFR1 (white histograms), P-VEGFR2 (grey histograms) and active caspase-3 (hatched histograms). **d** Apoptosis was evaluated by counting apoptotic MGH7 cells after Hoechst staining in *sVEGFR1-i13* (white bars) or *mismatch* (black bars) transfected cells. The mean ± SD of three independent experiments is shown. **e**, **f** The same experiments as in **b**, **c** were performed in H2170 cells. Statistical analyses (*p < 0.05, **p < 0.01, ***p < 0.001)

Next, we investigated the role of sVEGFR1-i13 in the response of SQLC cell lines to anti-angiogenic therapies. Of the SQLC cell lines, we first noticed that MGH7 cells are more resistant to anti-angiogenic agents than H2170, as shown by quantification of cell viability (Fig. 3a) as well as of colony formation (Supplementary Figure 3A). At the molecular level, a sustained activation of VEGFR1 and VEGFR2 was observed when MGH7 cells were treated for a long period of time with either bevacizumab or SU5416 (Supplementary Figure 3B). Same results were found in UN-SCC680 murine cells (Supplementary Figure 3C) and in SCC680 tumourgrafts (Supplementary Figure 3D) treated with anti-angiogenics. Importantly, the specific knock-down of sVEGFR1-i13 by using a mixture of two distinct siRNAs in MGH7 cells prevented the activation of VEGFR1/VEGFR2 proteins following bevacizumab or SU5416 treatment (Fig. 3b, c), and promoted apoptosis as detected by caspase-3 activation using western blotting (Fig. 3b, c) or Hoechst staining (Fig. 3d). Together and unexpectedly, these data suggested that accumulation of sVEGFR1-i13 protein contributes to the resistance of MGH7 tumour cells to anti-angiogenic therapies through sustained activation of VEGFR autocrine signalling pathways.

On the opposite, in H2170 cells which were more sensitive than MGH7 cells to anti-angiogenic therapies (Fig. 3a), bevacizumab or KI8751 significantly decreased VEGFR1 or VEGFR2 phosphorylation as expected (Fig. 3e, f and Supplementary Figure 3E), and induced apoptosis (Fig. 3e, f). Importantly, the knock-down of sVEGFR1-i13 in these cells prevented the decrease of VEGFR1/2 phosphorylation as well as caspase-3 activation upon treatment (Fig. 3e, f). As a whole, these results demonstrated that sVEGFR1-i13 regulates a VEGFR-dependent autocrine loop in SQLC tumour cells in response to anti-angiogenic therapies. However, depending on the cells, it mediates contrasting effects and contributes either to their resistance or to their sensitivity to these therapies.

### A cross-talk with β1 integrin is involved in the contrasting effects of sVEGFR1-i13 in response to anti-angiogenic therapies

**Fig. 4:**
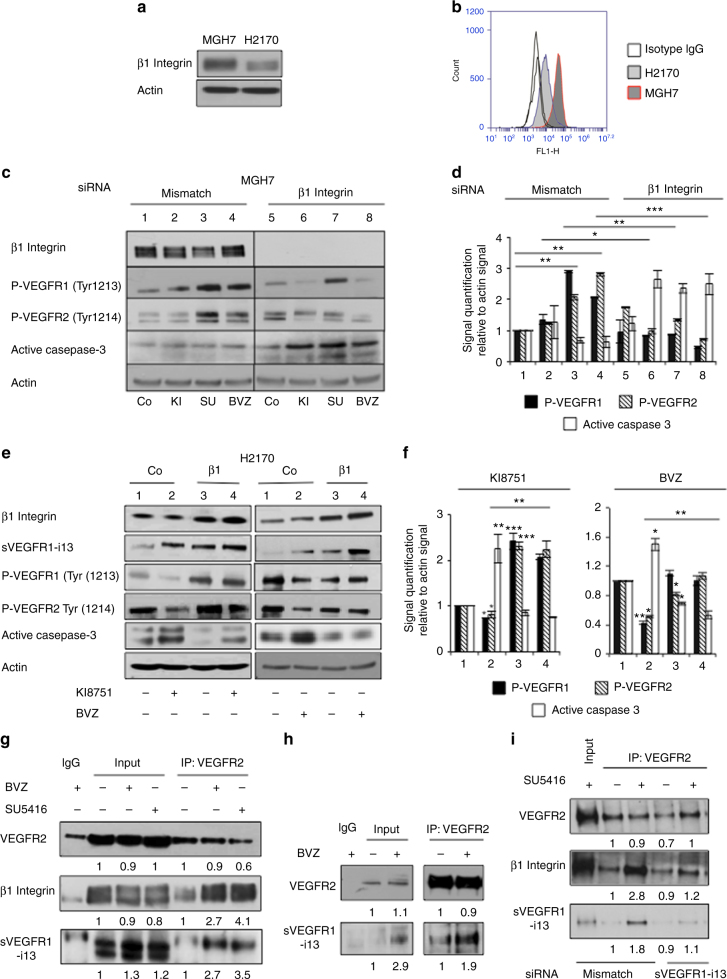
β1 integrin is a critical determinant of the contrasting effects of sVEGFR1-i13 in response to anti-angiogenic therapies in SQLC cells. **a** Western blot analysis for detection of β1 integrin was performed in MGH7 or H2170 cells as indicated. Actin was used as a loading control. **b** Flow cytometry analysis for the detection of β1 integrin was performed in non permeabilised MGH7 or H2170 cells. Irrelevant isotype IgG was used as a negative control. **c** Western blot experiments for the detection of the indicated proteins. MGH7 cells were transfected for 48 h with β*1 integrin* siRNA (or *mismatch* as indicated) and treated (or not - Co) for 24 additional hours with 10 µM KI8751 (KI) or 10 µM SU5416 (SU). For bevacizumab (BVZ), MGH7 cells were transfected during 72 h with β*1 integrin* (or *mismatch)* siRNA in the presence (or absence) of 10 µg/ml bevacizumab. Actin was used as a loading control. **d** Mean densitometric quantification of the indicated proteins relative to actin signal in 3 different experiments. Statistical analyses were performed using ANOVA test (*p < 0.05, **p < 0.01, ***p < 0.001). **e** Western blot analyses of the indicated proteins were performed in H2170 cells transfected for 48 h with a plasmid encoding β1 integrin (β1) and treated (+) or not (−) with 10 µM KI8751 for 24 additional hours (left panels) or 10 µg/ml bevacizumab for 72 h (right panels). Actin was used as a loading control. **f** Mean densitometric quantification of the indicated proteins relative to actin signal in 3 different experiments. Statistical analyses were performed using ANOVA test (*p < 0.05, **p < 0.01, ***p < 0.001). **g** VEGFR2 protein was immunoprecipitated using an anti-VEGFR2 antibody (clone 55Β11) from total protein extracts obtained from MGH7 cells that had been treated for 72 h with 10 µg/ml bevacizumab or for 24 h with 10 µM SU5416 as indicated. IgG was used as an irrelevant antibody. The presence of VEGFR2, β1 integrin or sVEGFR1-i13 protein in the immunoprecipitates was assessed by western blotting. Input represents 10% of the immunoprecipitates. **h** The presence of VEGFR2/sVEGFR1-i13 co-immunoprecipitates was assessed in H2170 cells that had been treated for 72 h with 10 µg/ml bevacizumab as mentioned in **e**. **i** VEGFR2 protein was immunoprecipitated in MGH7 cells transfected with *mismatch* or *sVEGFR1-i13* siRNA during 48 h and treated (+) or not (−) with 10 µM SU5416 for 24 additional hours. The presence of VEGFR2, β1 integrin or sVEGFR1-i13 protein in the immunoprecipitates was assessed by western blotting. Input represents 10% of the immunoprecipitates. **g–i** Numbers represent the quantification of VEGFR2, β1 integrin and sVEGFR1-i13 signal intensities using Image J software. In immunoprecipitation experiments, β1 integrin and sVEGFR1-i13 signals were determined according to VEGFR2 signal in each condition. The value 1 was arbitrarily assigned to the untreated condition signal

In endothelial cells, sVEGFR1-i13 together with β1 integrin activate several pathways such as PKC, Rac1 and VEGFR2 signalling, thereby promoting cell adhesion and migration.^14,15^ Interestingly, we showed that MGH7 cells express higher levels of β1 integrin compared to H2170 cells as depicted by western blotting (Fig. 4a) or flow cytometry (Fig. 4b). Therefore, we postulated that this differential expression level of β1 integrin could explain why sVEGFR1-i13 exhibits opposite effects in MGH7 or H2170 cells exposed to anti-angiogenic therapies.

In MGH7 cells, the knock-down of β1 integrin prevented the activation of VEGFR1 and VEGFR2 receptors upon treatment with SU5416 (Fig. 4c, d, compare lanes 3 and 7) or bevacizumab (Fig. 4c, d, compare lanes 4 and 8) and induced apoptosis. On the other hand, overexpressing β1 integrin in H2170 cells significantly reversed the decrease of P-VEGFR1(Tyr1213) and P-VEGFR2(Tyr1214) proteins upon treatment with KI8751 (Fig. 4e, f, compare lanes 2 and 4) or bevacizumab (Fig. 4e, f, compare lanes 6 and 8) and prevented the occurrence of apoptosis. Of note, anti-angiogenic treatments did not modify β1 integrin protein level in MGH7 (Fig. 4c) or H2170 (Fig. 4e) cells. Similar results were observed in murine UN-SCC680 and UN-ADC12 cells that both express β1 integrin (Supplementary Figure 4D). Using co-immunoprecipitation experiments, we further showed that bevacizumab and SU5416 stimulate the formation of a complex between VEGFR2, β1 integrin and sVEGFR1-i13 in MGH7 cells (Fig. 4g), whereas bevacizumab stimulated the formation of a complex between VEGFR2 and sVEGFR1-i13 proteins in H2170 cells (Fig. 4h). Proximity ligation assay in MGH7 cells treated with SU5416 confirmed the formation of VEGFR2/β1 integrin and VEGFR2/sVEGFR1-i13 complexes, which were detected at the plasma membrane and inside the cytoplasm (Supplementary Figures 4A-C). Importantly, the knock-down of sVEGFR1-i13 significantly prevented the formation of VEGFR2/β1 integrin complexes in SU5416-treated MGH7 cells (Fig. 4i), thereby indicating that sVEGFR1-i13 is required for VEGFR2/β1 integrin interaction in response to anti-angiogenic therapies. Overall, these results strongly suggest that a direct cross-talk with β1 integrin determines whether sVEGFR1-i13 positively or negatively regulates VEGFR signalling to promote either cell proliferation (resistance) or apoptosis (sensitivity) in response to anti-angiogenic therapies in SQLC tumour cell lines.

### The sVEGFR1-i13/β1 integrin cross-talk promotes cell proliferation in MGH7 cells and contributes to the progression of SQLC patients

**Fig. 5:**
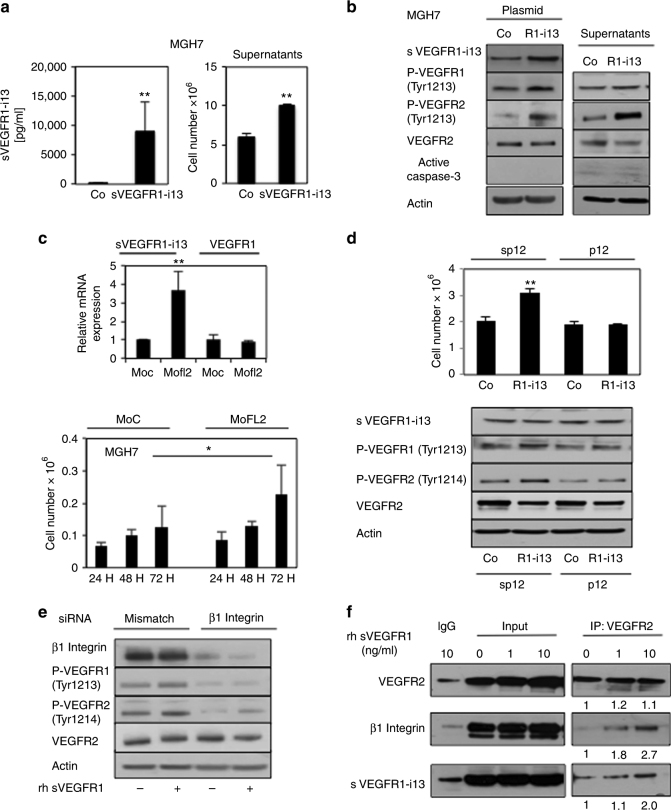
sVEGFR1-i13/β1 integrin autocrine cross-talk stimulates cell proliferation in MGH7 cells. **a**, **b** MGH7 cells were transfected and studied after 48 h with a plasmid encoding sVEGFR1-i13 (sVEGFR1-i13) or control, Co. **a** Left panels: quantification by ELISA of sVEGFR1-i13 in the supernatants. Right panels: cell number (x10^6^) was estimated following trypan blue staining in cells cultured for 48 h in the presence of the supernatants obtained from cells transfected with the plasmid encoding sVEGFR1-i13 (sVEGFR1-i13), or controls. The mean ± SD of three independent experiments is illustrated. **b** Western blot analyses of the indicated proteins in MGH7 cells either transfected (plasmid) with a plasmid encoding sVEGFR1-i13 (R1-i13) or control (Co), or cultured for 48 h in supernatants (supernatants) taken from cells transfected with a plasmid encoding sVEGFR1-i13 (R1-i13) or control (Co). Actin was used as a loading control. **c** MGH7 cells were transfected for the indicated times with either control (MoC) or sVEGFR1-i13 (MoFL2) morpholino. Cell number (x10^6^) was estimated following trypan blue staining. RT-qPCR in MGH7 cells demonstrated the efficiency of sVEGFR1-i13 morpholino as detected by the significant increase of *sVEGFR1-i13* mRNA level. The mean ± SD of three independent experiments is illustrated. **d** MGH7 cells were cultured for 72 h in the presence of supernatants taken from cells transfected for 48 h with a plasmid encoding sVEGFR1-i13 (R1-i13) or control (Co) in the presence of 10 µg/ml of either a scramble peptide (sp12) or a peptide blocking the interaction between sVEGFR1-i13 and β1 integrin (p12) as indicated. Upper panel: cell number (x10^6^) was estimated following trypan blue staining. Lower panel: western blot analyses of the indicated proteins were performed. Actin was used as a loading control. **e** Western blot analyses of the indicated proteins were performed in MGH7 cells transfected for 72 h with either *mismatch* or β*1 integrin* siRNA as indicated and treated (+) or not (−) for 24 h with 1 ng/ml sVEGFR1 recombinant ligand. Actin was used as a loading control. **f** VEGFR2 protein was immunoprecipitated using an anti-VEGFR2 antibody (clone 55Β11) from total protein extracts obtained from MGH7 cells treated or not for 24 h with 1 ng/ml sVEGFR1 recombinant ligand. IgG was used as an irrelevant antibody. The presence of VEGFR2, β1 integrin or sVEGFR1-i13 protein in the immunoprecipitates was assessed by western blotting. The ‘Input’ represents 10% of the amount used for the immunoprecipitations. Numbers represent the quantification of VEGFR2 and β1 integrin signal intensities in immunoprecipitates using Image J software. β1 integrin signals were determined according to VEGFR2 signal in each condition. The value 1 was arbitrarily assigned to the untreated condition signal. In all experiments, statistical analyses were performed using a student non paired *t-*test (**a**) or ANOVA test (**c, d**) (*p < 0.05, **p < 0.01)

Next, we asked whether the sVEGFR1-i13/β1 integrin/VEGFR signalling pathway could also control SQLC tumour cells proliferation in the absence of anti-angiogenics. To answer this question, we transfected MGH7 cells with a plasmid encoding sVEGFR1-i13. As a consequence, sVEGFR1-i13 was highly secreted in the cellular supernatants of these cells (Fig. 5a, left panel). Interestingly, naïve MGH7 cells cultured in presence of these conditioned supernatants proliferated faster than control cells (Fig. 5a, right panel). At the molecular level, as compared to control cells, accumulation of P-VEGFR1(Tyr1213) and P-VEGFR2(Tyr1214) proteins was observed in MGH7 transfected cells (plasmid), as well as in naïve MGH7 cells cultured in conditioned supernatants (supernatants) (Fig. 5b). To confirm the role of sVEGFR1-i13 in cell proliferation, MGH7 cells were transfected with a splice site-blocking morpholino which induces a switch from full-length *VEGFR1* to *sVEGFR1-i13* mRNA^22^ (Fig. 5c, upper panel). Re-directing the splicing toward sVEGFR1-i13 increased cell proliferation (Fig. 5c, lower panel). As a whole, these results were consistent with an autocrine function of sVEGFR1-i13 in triggering VEGFR activation and cell proliferation in MGH7 cells. To determine whether β1 integrin was involved, we used a synthetic NYLTHRQ peptide (p12) that blocks the interaction between sVEGFR1-i13 and β1 integrin in endothelial cells.^23^ Activation of VEGFRs and increased proliferation were prevented when this peptide was added to conditioned supernatants put onto naïve MGH7 cells (Fig. 5d). In the same conditions, a scramble LTQNYRH peptide (sp12) had no inhibitory effect. These results showed that the interaction between sVEGFR1-i13 and β1 integrin contributes to VEGFRs activation. In agreement with these data, recombinant sVEGFR1 stimulated MGH7 colony formation in soft agar assays (data not shown) and activated VEGFR1/VEGFR2 in the same way as the sVEGFR1-13 encoding plasmid (Fig. 5e). Importantly, the knock-down of β1 integrin by RNA interference prevented VEGFR1/2 activation (Fig. 5e). In addition, recombinant sVEGFR1 increased the formation of VEGFR2/β1 integrin complexes (Fig. 5f).

Opposing results on cell proliferation, colony formation and VEGFR1/2 activation were obtained in H2170 cells cultured in the presence of conditioned supernatants (Supplementary Figures 5A and 5B) or transfected either with a sVEGFR1-i13-encoding plasmid (Supplementary Figure 5B), MoFL2 morpholinos (Supplementary Figure 5C) or treated with recombinant sVEGFR1 (Supplementary Figure 5D). In addition, recombinant sVEGFR1 stimulated the formation of a complex between VEGFR2 and sVEGFR1-i13 in these cells (Supplementary Figure 5E). As a whole, these data indicate that sVEGFR1-i13 exhibits contrasting functions in SQLC tumour cells by promoting either proliferation or apoptosis, as it does in the presence of angiogenic therapies. Importantly, such a contrasting response also involves its cross-talk with β1 integrin.

**Fig. 6:**
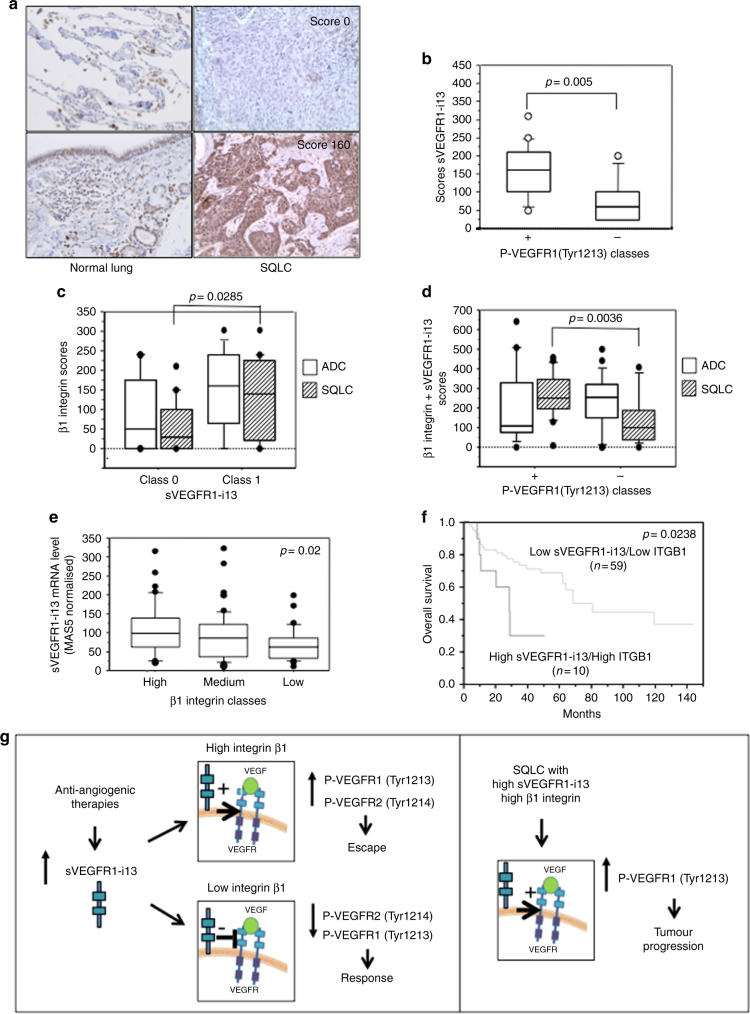
sVEGFR1-i13/β1 integrin cross-talk is involved in SQLC tumour progression. **a** Representative sVEGFR1-i13 immunostainings from paraffin-embedded sections of normal lung tissues (upper panel: alveolus; lower panel: bronchus) as well as of two well-differentiated squamous cell lung carcinomas. Scores are indicated for each case. **b** Mean levels ± SD of sVEGFR1-i13 immunohistochemical scores according to the P-VEGFR1(Tyr1213) status in squamous cell lung carcinoma, where + and – represent tumours with high or low levels of P-VEGFR1(Tyr1213) compared to normal lung tissues respectively. Statistical analyses were performed using a non parametric Mann–Whitney test. **c** Mean levels ± SD of β1 integrin immunohistochemical scores according to the sVEGFR1-i13 status in ADC (white boxes) and SQLC (hatched boxes), where + and – represent tumours with high or low levels of sVEGFR1-i13 compared to normal lung tissues respectively. Statistical analyses were performed using a non parametric Mann–Whitney test. **d** Mean levels ± SD of β1 integrin+sVEGFR1-i13 immunohistochemical scores according to P-VEGFR1(Tyr1213) status in ADC (white boxes) and SQLC (hatched boxes), where + and – represent tumours with high or low levels of P-VEGFR1(Tyr1213) compared to normal lung tissues respectively. Statistical analyses were performed using a non parametric Mann–Whitney test. **e** Mean levels ± SD of MAS5-normalised *sVEGFR1-i13* mRNA in SQLC patients taken from the GSE4573 database expressing either low (<25th percentile), medium (25–75th percentile) or high (>75th percentile) levels of β*1 integrin (ITGB1)* mRNA. Statistical analyses were performed using Kruskal–Wallis test. **f** Kaplan–Meier univariate survival analysis of SQLC patients with pTNM I/II stage according to high (>75th percentile; 4th quartile) or low/medium (<75th percentile; 1st–3rd quartiles) *sVEGFR1-i13* and β*1 integrin (ITGB1)* mRNA levels. **h** Left panels: anti-angiogenic therapies increase the level of sVEGFR1-i13 in squamous lung tumour cells. However, whether cells respond or do not respond to anti-angiogenic therapies depends on the level of β1 integrin. In cells with high levels of β1 integrin (MGH7-like phenotype), the increase of sVEGFR1-i13 protein level upon anti-angiogenic treatment contributes to the activation of a β1 integrin-dependent VEGFR1/VEGFR2 autocrine loop. This loop promotes the tumour cell proliferation and survival, thereby escape from the treatment. In contrast, in cells with low β1 integrin expression (H2170-like phenotype), sVEGFR1-i13 heterodimerises with VEGFRs, prevents VEGFR signalling and induces apoptosis. We propose that this H2170-like phenotype might represent a subclass of SQLC patients who could be more responsive to ramucirumab treatment than the others. Right panel: In the absence of anti-angiogenic therapies, in SQLC tumours expressing high expression level of β1 integrin, the sVEGFR1-i13/β1 integrin/VEGFR cross-talk might contribute to the progression of squamous lung carcinoma patients

To date, nothing is known regarding the expression level of sVEGFR1-i13 in NSCLC. Therefore, we performed an immunohistochemical analysis of sVEGFR1-i13 in a retrospective cohort of NSCLC tumour samples using the sVEGFR1-i13 antibody. In normal lung taken at distance from the tumour site, moderate diffuse cytoplasmic and nuclear staining of sVEGFR1-i13 was seen in hyperplastic type II pneumonocytes and bronchial basal cells (Fig. 6a). Compared to normal lung tissues, SQLC and ADC patients had a heterogenous pattern of sVEGFR1-i13 expression (Fig. 6a, Supplementary Figures 6A, B, Table 1). Interestingly, an association between high scores of sVEGFR1-i13 and advanced (pTNM – T-size, N-lymph node, M-metastasis) stages was observed in SQLC but not in ADC patients (Supplementary Table 2, *p* = 0.05). Importantly, when we analysed the expression of P-VEGFR1(Tyr1213) by immunohistochemistry in the same samples, we found that sVEGFR1-i13 and P-VEGFR1(Tyr1213) immunostainings correlate in SQLC tumours (Fig. 6b, *p* = 0.005). Unfortunately, we were not able to assess the status of P-VEGFR2(Tyr1214) as the antibody did not work in immunohistochemistry. β1 integrin expression was studied in the same series of tumour samples (Supplementary Table 3). We found that β1 integrin scores positively correlated with sVEGFR1-i13 classes in SQLC patients only (Fig. 6c, *p* = 0.0285). In addition, high levels of both β1 integrin and sVEGFR1-i13 proteins were associated with P-VEGFR1(Tyr1213) immunostaining (Fig. 6d, *p* = 0.0036) and advanced III/IV pTNM stages (Supplementary Table 4, *p* = 0.05) in SQLC but not in ADC patients. These data strongly suggested that sVEGFR1-i13 together with β1 integrin stimulate VEGFR1 phosphorylation in SQLC tumours, as observed in MGH7 cells, and promote a more aggressive phenotype. Consistently, an increased immunostaining of sVEGFR1-i13 or β1 integrin was depicted in clusters of migrating SQLC tumour cells as well as in tumour cells localised at the invasive front (Supplementary Figures 6B and D).

To further analyse the relationship between sVEGFR1-i13 and β1 integrin, we took advantage of an Affymetrix dataset published in a cohort of 130 SQLC patients which contained two probe sets that distinguish between *sVEGFR1-i13* and full-length *VEGFR1* mRNAs [Gene Omnibus data set GSE4573^24^]. When SQLC patients were sub-divided in quartiles according to *VEGFR1* and *sVEGFR1-i13* mRNA levels, we found that 33% of patients with low *sVEGFR1-i13* mRNA had high *VEGFR1* mRNA, whereas 18% of patients with high *sVEGFR1-i13* mRNA showed low *VEGFR1* mRNA (Supplementary Figure 6E, *p* = 0.03). These data indicated that *sVEGFR1-i13* and full-length *VEGFR1* mRNAs levels are inversely associated in these patients which was consistent with pre-mRNA alternative splicing of VEGFR1. In addition, when SQLC patients were sub-divided in three classes according to their β*1 integrin* mRNA levels (high, medium, low expression), a correlation between β*1 integrin* and *sVEGFR1-i13* mRNA expression levels was found (Fig. 6e, *p* = 0.02). This was confirmed using a Spearman correlation test (*p* = 0.0028, *r* = 0.263; data not shown) and in another cohort including 135 SQLC patients (Gene Omnibus data set GSE68793; Supplementary Figure 6F). Importantly, high levels of both *sVEGFR1-i13* and β*1 integrin* mRNAs (>75th percentile; 4th quartile) were associated with poor prognosis in SQLC patients with early pTNM stages (from the GSE4573 cohort) (Fig. 6f, *p* = 0.0238), while no significance was observed when each marker alone was considered (data not shown). Taken together, these results indicated that the sVEGFR1-i13/β1 cross-talk contributes to the progression of squamous lung carcinoma patients.

## Discussion

Various studies have demonstrated a correlation between high sVEGFR1 plasma/serum levels and a poor response to anti-angiogenic therapies in breast, colon, rectal or advanced hepatocellular carcinoma. This poor response was associated with the dysregulation of vascular function as evidenced by insufficient vascular network or decreased vascular permeability.^12,25–29^ In this study, we highlight a new role for a sVEGFR1 splice variant in cancer. We demonstrate that sVEGFR1-i13 either inhibits or activates VEGFR1/VEGFR2 signalling in squamous lung carcinoma cells, depending on its cross-talk with β1 integrin. Importantly, we demonstrate that the sVEGFR1-i13/β1 integrin/VEGFRs autocrine loop is a critical determinant of the response, proliferation versus apoptosis, of squamous lung carcinoma cell lines to anti-angiogenic therapies, as well as is involved in the progression of squamous lung carcinoma patients.

The mechanisms that regulate the expression of VEGFR1 splice variants in cancerous cells are largely unknown. Several clinical trials reported variations in circulating levels of sVEGFR1 following anti-angiogenic therapies,^12,26–29^ but none of them investigated intra-tumoural levels, or pointed out to a specific mechanism for sVEGFR1 generation. Here we show that anti-angiogenic therapies increase the intra-tumoural expression level of the sVEGFR1-i13 splice variant in squamous cell lung carcinoma. Interestingly, we did not observe similar variations of the other VEGFR1 splice variants in the same conditions. In endothelial cells, it was previously shown that the expression of sVEGFR1-i13 is regulated by VEGF-A,^30,31^ hypoxia^32^ or by the oxygen-sensing hydroxylase JMJD6 together with the splicing factor U2AF65.^33,34^ We did not find any significant effect of hypoxia or JMJD6 on sVEGFR1-i13 expression, in control or treated cells in our cellular models (data not shown). However, we obtained evidence of a correlation between sVEGFR1-i13 and β1 integrin expression levels in SQLC cell lines (data not shown) and primary tumours (Fig. 6e and Supplementary Figure 6F). Therefore, the relationship between β1 integrin signalling and *VEGFR1* RNA splicing warrants further deepening.

We provide here the first description of sVEGFR1-i13 protein expression in NSCLC patients. We find that high levels of both sVEGFR1-i13 and β1 integrin proteins correlate with high levels of P-VEGFR1(Tyr1213) as well as with advanced pTNM stages in SQLC patients only (not in ADC patients). Moreover, we show that SQLC patients with early pTNM stages and high levels of both *sVEGFR1-i13* and β*1 integrin* mRNAs have poor outcome. These results are consistent with the data we obtained in MGH7 cells showing pro-proliferative and pro-survival effects of sVEGFR1-i13 through activation of β1 integrin/VEGFR signalling pathway. The role of sVEGFR1-i13 as an actor of tumour progression was unexpected since sVEGFR1 is generally considered to be an anti-angiogenic factor because it sequesters VEGF-A and forms inactive heterodimers with VEGFR1 or VEGFR2.^5,7^ However, other studies have challenged the simplistic view of sVEGFR1 being only an anti-angiogenic factor by demonstrating that sVEGFR1-i13 stimulates endothelial cell adhesion and migration through its interaction with α5β1 integrin.^14^ In addition, a recent study showed that sVEGFR1 also acts on tumour cells themselves to promote non-apoptotic death.^16^ In light of our data, we now propose a new tumoural role for the sVEGFR1-i13 splice variant (Fig. 6g), whereby its cross-talk with β1 integrin determines the activation/inhibition of the VEGFR signalling pathway to control SQLC tumour cells fate (proliferation versus apoptosis) in response to anti-angiogenic therapies as well as during the progression of SQLC malignancy.

Squamous cell lung carcinoma patients are not eligible for bevacizumab treatment because it often leads to fatal haemorragies, which are not observed in lung ADC patients treated with the same therapies. Bevacizumab-treated SQLC patients also suffer hypertension and proteinuria, which are clinical symptoms similar to those of preeclampsia, a pregnancy disorder in which overproduction of sVEGFR1 is a major determinant of the vascular dysfunction.^35^ Therefore, from a clinical point of view, our observation that anti-angiogenic therapies up-regulate sVEGFR1-i13 in SQLC but not in ADC tumours might offer a plausible explanation for the adverse effects of anti-angiogenic therapies in SQLC patients. Identification of the molecular determinants of the response to anti angiogenic therapies is of critical importance in SQLC. Hence, the anti-VEGFR2 antibody, ramucirumab, was recently approved for second-line therapy in stage IV SQLC^36^ and there is no predictive biomarker to select patients who will benefit from this therapy. Based on our results, one might speculate that SQLC patients with low levels of sVEGFR1-i13 and β1 integrin would be better responders than those expressing high basal levels. Interestingly, it has been previously shown that OS2966, an inhibitory β1 integrin antibody, potentiates bevacizumab treatment in bevacizumab-resistant human glioma.^37,38^ Therefore, another interesting prediction based on our results is that anti-angiogenic agents might synergise with β1 integrin antagonists in SQLC patients. Overall, our results pave the way for new therapeutic opportunities and predictive biomarkers in squamous cell lung carcinoma patients for whom the therapeutic arsenal remains poor compared to that for lung ADC patients.

## Electronic supplementary material

Supplementary Figure Legends

Supplementary Figure 1

Supplementary Figure 2

Supplementary Figure 3

Supplementary Figure 4

Supplementary Figure 5

Supplementary Figure 6

Supplementary Table 1

Supplementary Table 2

Supplementary Table 3

Supplementary Table 4

## Availability of data and materials

GSE4573 and GSE68793 datasets used in this study were recovered from Gene Omnibus.

## References

[1] Heist RS, Sequist LV, Engelman JA (2012). Genetic changes in squamous cell lung cancer: a review. J. Thorac. Oncol..

[2] Larrayoz M (2014). Contrasting responses of non-small cell lung cancer to antiangiogenic therapies depend on histological subtype. EMBO Mol. Med..

[3] Johnson DH (2004). Randomized phase II trial comparing bevacizumab plus carboplatin and paclitaxel with carboplatin and paclitaxel alone in previously untreated locally advanced or metastatic non-small-cell lung cancer. J. Clin. Oncol..

[4] Scagliotti G, Govindan R (2010). Targeting angiogenesis with multitargeted tyrosine kinase inhibitors in the treatment of non-small cell lung cancer. Oncologist.

[5] Kendall RL, Thomas KA (1993). Inhibition of vascular endothelial cell growth factor activity by an endogenously encoded soluble receptor. Proc. Natl Acad. Sci. USA.

[6] Wu FT (2010). A systems biology perspective on sVEGFR1: its biological function, pathogenic role and therapeutic use. J. Cell. Mol. Med..

[7] Kendall RL, Wang G, Thomas KA (1996). Identification of a natural soluble form of the vascular endothelial growth factor receptor, FLT-1, and its heterodimerization with KDR. Biochem. Biophys. Res. Commun..

[8] Goldman CK (1998). Paracrine expression of a native soluble vascular endothelial growth factor receptor inhibits tumor growth, metastasis, and mortality rate. Proc. Natl Acad. Sci. USA.

[9] Owen LA (2012). Morpholino-mediated increase in soluble Flt-1 expression results in decreased ocular and tumor neovascularization. PLoS One.

[10] Bando H (2005). Association between intratumoral free and total VEGF, soluble VEGFR-1, VEGFR-2 and prognosis in breast cancer. Br. J. Cancer.

[11] Lambrechts D, Lenz HJ, de Haas S, Carmeliet P, Scherer SJ (2011). Markers of response for the antiangiogenic agent bevacizumab. J. Clin. Oncol..

[12] Tolaney SM (2015). Role of vascular density and normalization in response to neoadjuvant bevacizumab and chemotherapy in breast cancer patients. Proc. Natl Acad. Sci. USA.

[13] Heist RS (2015). Improved tumor vascularization after anti-VEGF therapy with carboplatin and nab-paclitaxel associates with survival in lung cancer. Proc. Natl Acad. Sci. USA.

[14] Orecchia A (2003). Vascular endothelial growth factor receptor-1 is deposited in the extracellular matrix by endothelial cells and is a ligand for the alpha 5 beta 1 integrin. J. Cell. Sci..

[15] Orecchia A (2014). Endothelial cell adhesion to soluble vascular endothelial growth factor receptor-1 triggers a cell dynamic and angiogenic phenotype. FASEB J..

[16] Miyake T (2016). Soluble VEGF receptor 1 (sFLT1) induces non-apoptotic death in ovarian and colorectal cancer cells. Sci. Rep..

[17] Merdzhanova G (2008). E2F1 controls alternative splicing pattern of genes involved in apoptosis through upregulation of the splicing factor SC35. Cell Death Differ..

[18] Ruffini F (2011). Expression of the soluble vascular endothelial growth factor receptor-1 in cutaneous melanoma: role in tumour progression. Br. J. Dermatol..

[19] Travis WD (2011). International association for the study of lung cancer/american thoracic society/european respiratory society international multidisciplinary classification of lung adenocarcinoma. J. Thorac. Oncol..

[20] Gout S (2012). Abnormal expression of the pre-mRNA splicing regulators SRSF1, SRSF2, SRPK1 and SRPK2 in non small cell lung carcinoma. PLoS One.

[21] Salon C (2006). E2F1 induces apoptosis and sensitizes human lung adenocarcinoma cells to death-receptor-mediated apoptosis through specific downregulation of c-FLIP(short). Cell Death Differ..

[22] Vorlova S (2011). Induction of antagonistic soluble decoy receptor tyrosine kinases by intronic polyA activation. Mol. Cell.

[23] Soro S (2008). A proangiogenic peptide derived from vascular endothelial growth factor receptor-1 acts through alpha5beta1 integrin. Blood.

[24] Raponi M (2006). Gene expression signatures for predicting prognosis of squamous cell and adenocarcinomas of the lung. Cancer Res..

[25] Thomas-Schoemann A (2015). Soluble VEGFR-1: a new biomarker of sorafenib-related hypertension (i.e., sorafenib-related is the compound adjective?). J. Clin. Pharmacol..

[26] Meyerhardt JA (2012). Phase I study of cetuximab, irinotecan, and vandetanib (ZD6474) as therapy for patients with previously treated metastastic colorectal cancer. PLoS One.

[27] Duda DG (2010). Plasma soluble VEGFR-1 is a potential dual biomarker of response and toxicity for bevacizumab with chemoradiation in locally advanced rectal cancer. Oncologist.

[28] Willett CG (2009). Efficacy, safety, and biomarkers of neoadjuvant bevacizumab, radiation therapy, and fluorouracil in rectal cancer: a multidisciplinary phase II study. J. Clin. Oncol..

[29] Zhu AX (2013). A phase II and biomarker study of ramucirumab, a human monoclonal antibody targeting the VEGF receptor-2, as first-line monotherapy in patients with advanced hepatocellular cancer. Clin. Cancer Res..

[30] Ahmad S (2011). Autocrine activity of soluble Flt-1 controls endothelial cell function and angiogenesis. Vasc. Cell.

[31] Saito T (2013). VEGF-A induces its negative regulator, soluble form of VEGFR-1, by modulating its alternative splicing. FEBS Lett..

[32] Thomas CP (2009). A recently evolved novel trophoblast-enriched secreted form of fms-like tyrosine kinase-1 variant is up-regulated in hypoxia and preeclampsia. J. Clin. Endocrinol. Metab..

[33] Boeckel JN (2011). Jumonji domain-containing protein 6 (Jmjd6) is required for angiogenic sprouting and regulates splicing of VEGF-receptor 1. Proc. Natl Acad. Sci. USA.

[34] Palmer KR (2016). Jumonji domain containing protein 6 is decreased in human preeclamptic placentas and regulates sFLT-1 splice variant production. Biol. Reprod..

[35] Jain RK (2005). Normalization of tumor vasculature: an emerging concept in antiangiogenic therapy. Science.

[36] Garon EB (2014). Ramucirumab plus docetaxel versus placebo plus docetaxel for second-line treatment of stage IV non-small-cell lung cancer after disease progression on platinum-based therapy (REVEL): a multicentre, double-blind, randomised phase 3 trial. Lancet.

[37] Carbonell WS, DeLay M, Jahangiri A, Park CC, Aghi MK (2013). beta1 integrin targeting potentiates antiangiogenic therapy and inhibits the growth of bevacizumab-resistant glioblastoma. Cancer Res..

[38] Jahangiri A, Aghi MK, Carbonell WS (2014). beta1 integrin: critical path to antiangiogenic therapy resistance and beyond. Cancer Res..

