# Supplementary material for: The sVEGFR1-i13 splice variant regulates a β1 integrin/VEGFR autocrine loop involved in the progression and the response to anti-angiogenic therapies of squamous cell lung carcinoma
Source: Br J Cancer. 2018 May 24;118(12):1596–608. doi: 10.1038/s41416-018-0128-4 (PMC6008445; doi:10.1038/s41416-018-0128-4)
Supplement: Supplementary file 1 — Supplementary Figure Legends [file 41416_2018_128_MOESM1_ESM.docx]

**Supplementary Figure Legends**

**Supplementary Figure 1.** **Anti-angiogenic treatments do not regulate other VEGFR1 splice variants.** (A) Schematic representation of the full-length *VEGFR1* transcript as well as of the different *sVEGFR1* splice variants. (B, C) RT-qPCR analyses for the specific detection of *sVEGFR1-e15a* and *sVEGFR1-i14* transcripts, together with the full-length *VEGFR1* transcript in MGH7 (B) or H2170 (C) cells, treated, or not (Co), with 10µM SU5416 (SU) or 10µM KI8751 (KI) for 24 hours or with 10µg/ml bevacizumab (BVZ) for 72 hours as indicated. *GAPDH* was used as an internal control. Statistical analyses were performed using ANOVA test (* p<0.05).

**Supplementary Figure 2. sVEGFR1-i13 does not accumulate in lung adenocarcinoma cell lines treated with anti-angiogenic therapies.** (A, B, C) H358, H1299 and A549 lung adenocarcinoma cell lines were treated or not (Co) for 24 hours with 10µM KI8751 or 10µM SU5416 or for 72 hours with 10µg/ml bevacizumab (BVZ) as indicated. (A) Extra-cellular levels of sVEGFR1 in the supernatants were quantified by ELISA. (B) Western blot analysis was performed for the detection of sVEGFR1-i13. Actin was used as a loading control. (C) RT-qPCR analyses of *VEGFR1* (black bars) or *sVEGFR1-i13* (white bars) mRNA level were performed. *GAPDH* was used as an internal control. (D, E) Murine squamous cell lung carcinoma UN-SCC680 (D) or lung adenocarcinoma UN-ADC12 (E) cells were treated with the indicated concentrations of KI8751 or SU5416 for 24 hours. Western blot analysis of sVEGFR1-i13 was performed. Actin was used as a loading control. Histograms represent the quantification of the specific signal relative to actin in at least 3 different experiments. In all experiments, statistical analyses were performed using ANOVA or Student t test (* p<0.05; ** p<0.01, *** p<0.001).

**Supplementary Figure 3. Identification of two differential patterns of response to anti-angiogenic therapies.** (A) *Upper panels:* Clonogenic assays were performed in MGH7 and H2170 cells treated or not for 30 days with the indicated concentrations of bevacizumab (BVZ), KI8751 or SU5416 as indicated. *Lower panels:* crystal violet staining was dissolved in DMSO and DO was measured at 570 nm. Histogramms represent the mean±SD of three independent experiments performed in triplicate. Statistical analyses were performed using ANOVA test (* p<0.05, ** p<0.01). (B) Western blot analyses were performed for the detection of the indicated proteins in MGH7 cells treated or not for 72 hours with bevacizumab (BVZ, left panels), or for 24 hours with the indicated concentrations (µM) of SU5416 (right panels). Actin was used as a loading control. (C) Western-blotting of the indicated proteins in UN-SCC680 murine cells treated or not with the indicated concentrations (µM) of SU5416 for 24 hours. Actin was used as a loading control. (D) Immunohistochemical staining was performed on paraffin-embedded sections obtained from the same SQLC tumorgraft experiments as in Figure 2 having received or not sunitinib. Representative immunostainings of P-VEGFR1(Tyr1213) are presented. Two examples are illustrated for each condition. (E) Western blot analyses were performed for the detection of the indicated proteins in H2170 cells treated or not during 72 hours with bevacizumab (BVZ, left panels), or during 24 hours with the indicated concentrations (µM) of KI8751 (right panels). Actin was used as a loading control.

**Supplementary Figure 4. Anti-angiogenic therapies regulate the formation of β1 integrin/VEGFR2/sVEGFR1-i13 complexes.** (A, B) Proximity Ligation Assay was performed for endogenous β1 integrin and VEGFR2 (A) or endogenous sVEGFR1-i13 and VEGFR2 (B) proteins in MGH7 cells treated or not with 10µM SU5416 for 24 hours or 10 µg/ml BVZ for 72 hours. Scale bar = 10µm. (C) Quantification of β1 integrin and VEGFR2 (left panel) or sVEGFR1-i13 and VEGFR2 (right panel) PLA interactions was made from three independent experiments using ICY software and spot detector (n = 50 cells each, mean ± SD, Student’s t-test, two-tailed). (D) Western blot analyses were performed for the detection of β1 integrin in UN-SCC680 and UN-ADC12 cells treated or not for 24 hours with 10 µM KI8751 or SU5416. Tubulin was used as a loading control.

**Supplementary Figure 5. sVEGFR1-i13 inhibits cell proliferation of H2170 cells.**

(A) H2170 cells were transfected and studied after 48 hours with a plasmid encoding sVEGFR1-i13 (sVEGFR1-i13) or control, Co. *Left panels:* quantification by ELISA of sVEGFR1-i13 in the supernatants. *Right panels:* cell number (x10^6^) was estimated following trypan blue staining in cells cultured for 48 hours in the presence of the supernatants obtained from cells transfected with the plasmid encoding sVEGFR1-i13 (sVEGFR1-i13), or controls. The mean ± SD of three independent experiments is illustrated. (B) Western blot analyses of the indicated proteins in H2170 cells either transfected (plasmid) with a plasmid encoding sVEGFR1-i13 (R1-i13) or control (Co) or cultured for 48 hours in supernatants (supernatants) taken from cells transfected with a plasmid encoding sVEGFR1-i13 (R1-i13) or control (Co) as indicated. Actin was used as a loading control. (C) H2170 cells were transfected for the indicated times with either control (MoC) or sVEGFR1-i13 (MoFL2) morpholino. Cell number (x10^6^) was estimated following trypan blue staining. The mean ± SD of three independent experiments is illustrated. (D) Western blot analyses of the indicated proteins were performed in H2170 cells treated (+) or not (-) for 24 hours with 1 ng/ml sVEGFR1 recombinant ligand. Actin was used as a loading control. (E) VEGFR2 protein was immunoprecipitated using an anti-VEGFR2 antibody (clone 55B11) from total protein extracts obtained from H2170 cells treated or not for 24 hours with 1 ng/ml sVEGFR1 recombinant ligand. IgG was used as an irrelevant antibody. The presence of VEGFR2 or sVEGFR1-i13 protein in the immunoprecipitates was assessed by western blotting. The ‘Input’ represents 10% of the amount used for the immunoprecipitations. Numbers represent the quantification of VEGFR2 and sVEGFR1-i13 signal intensities in immunoprecipitates using Image J software. The value 1 was arbitrarily assigned to the untreated condition signal. In all experiments, statistical analyses were performed using a student non paired t test (A) or ANOVA test (C) (** p<0.01).

**Supplementary Figure 6. sVEGFR1-i13 and β1 integrin are expressed at different levels in NSCLC.** (A) Distribution of sVEGFR1-i13 immunostaining scores in squamous cell lung carcinoma (upper panel) or lung adenocarcinoma (lower panel) patients. (B) Representative sVEGFR1-i13 immunostainings from paraffin-embedded sections of two keratinizing squamous lung carcinoma (upper panels) and lung adenocarcinoma (lower panels). Scores are indicated for each case. Of note, in squamous lung carcinoma, high levels of sVEGFR1-i13 were observed in clusters of migrating tumor cells (a) and in tumor cells at the invasive front (b). (C) Distribution of β1 integrin immunostaining scores in squamous cell lung carcinoma and lung adenocarcinoma patients. (D) Representative β1 integrin immunostainings from paraffin-embedded sections of squamous lung carcinoma. Scores are indicated for each case. (a) non keratinizing SQLC with stroma negative for β1 integrin staining and strongly positive tumor cells invading alveolar lumens at the edge of the tumor front; (b) keratinizing SQLC with both tumor and stroma expressing β1 integrin; (c) non keratinizing SQLC with clusters of migrating tumor cells highly stained for β1 integrin; (d) keratinizing SQLC with tumor cells stained for β1 integrin at the invasive front. (E) Percentage of SQLC patients from the GSE4573 database with low (< 25^th^ percentile; white bars), medium (25^th^-75^th^ percentile, grey bars) or high (>75^th^ percentile, black bars) full-length *VEGFR1* mRNA levels according to their level of expression of *sVEGFR1-i13* mRNA (low expression < 25^th^ percentile; medium 25^th^-75^th^ percentile; high >75^th^ percentile). Statistical analyses were performed using a Chi2 test. (D) Mean levels ± standard deviation of MAS5-normalized *sVEGFR1-i13* mRNA in SQLC patients from the GSE68793 database expressing either low (<25^th^ percentile), medium (25^th^-75^th^ percentile) or high (>75^th^ percentile) levels of *β1 integrin (ITGB1)* mRNA. Statistical analyses were performed using Kruskal-Wallis test.
