# Supplementary figures and images for: The sVEGFR1-i13 splice variant regulates a β1 integrin/VEGFR autocrine loop involved in the progression and the response to anti-angiogenic therapies of squamous cell lung carcinoma
Source: Br J Cancer. 2018 May 24;118(12):1596–608. doi: 10.1038/s41416-018-0128-4 (PMC6008445; doi:10.1038/s41416-018-0128-4)

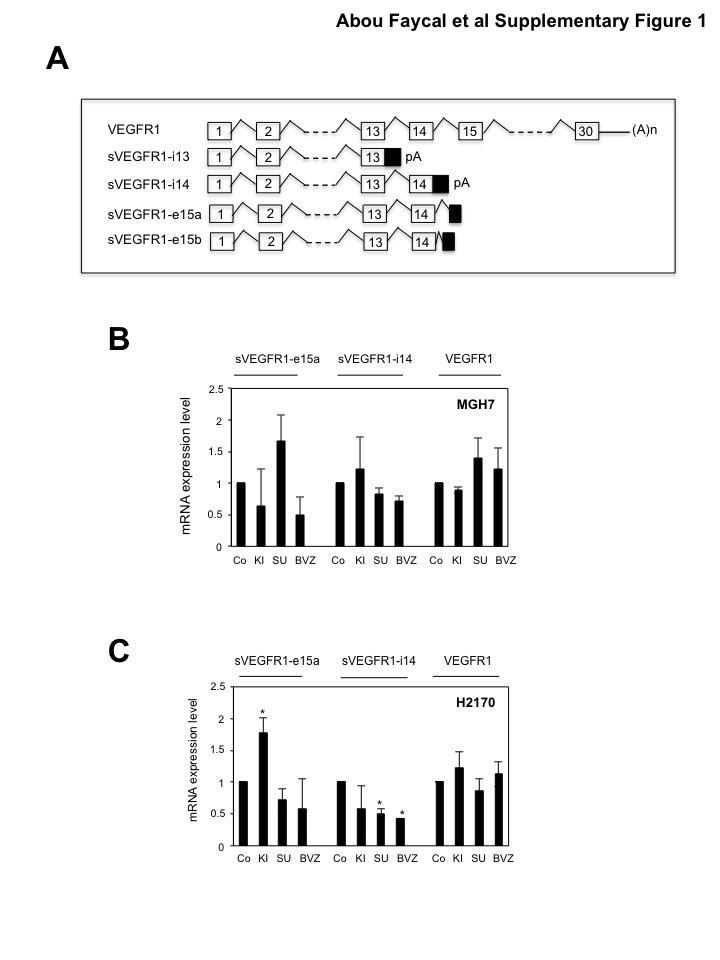

Supplement: Supplementary file 2 — Supplementary Figure 1 [file 41416_2018_128_MOESM2_ESM.tif]

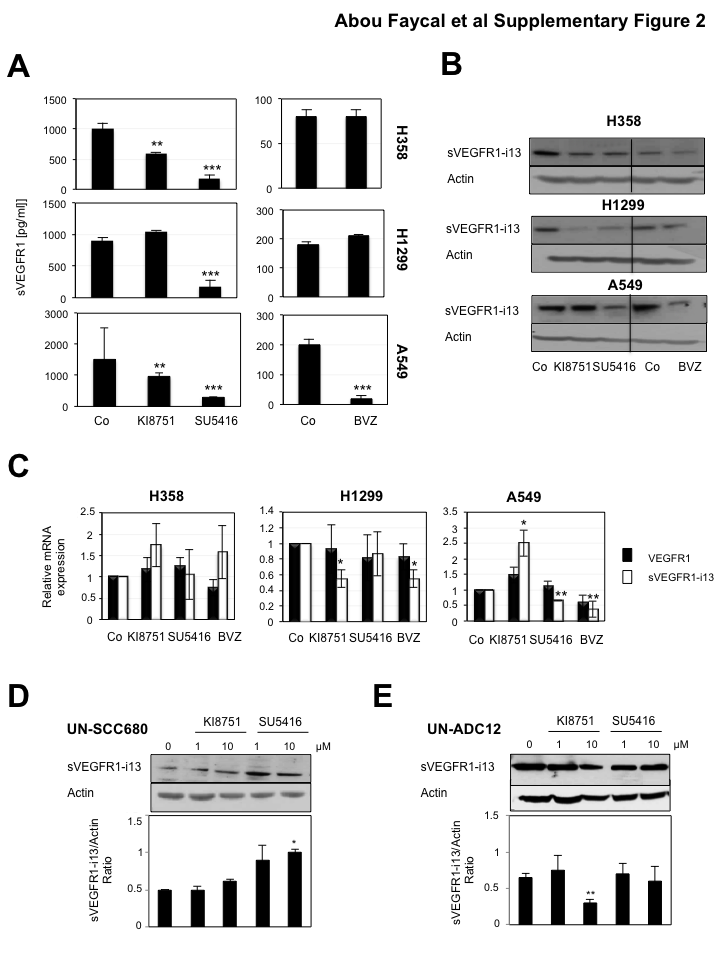

Supplement: Supplementary file 3 — Supplementary Figure 2 [file 41416_2018_128_MOESM3_ESM.tif]

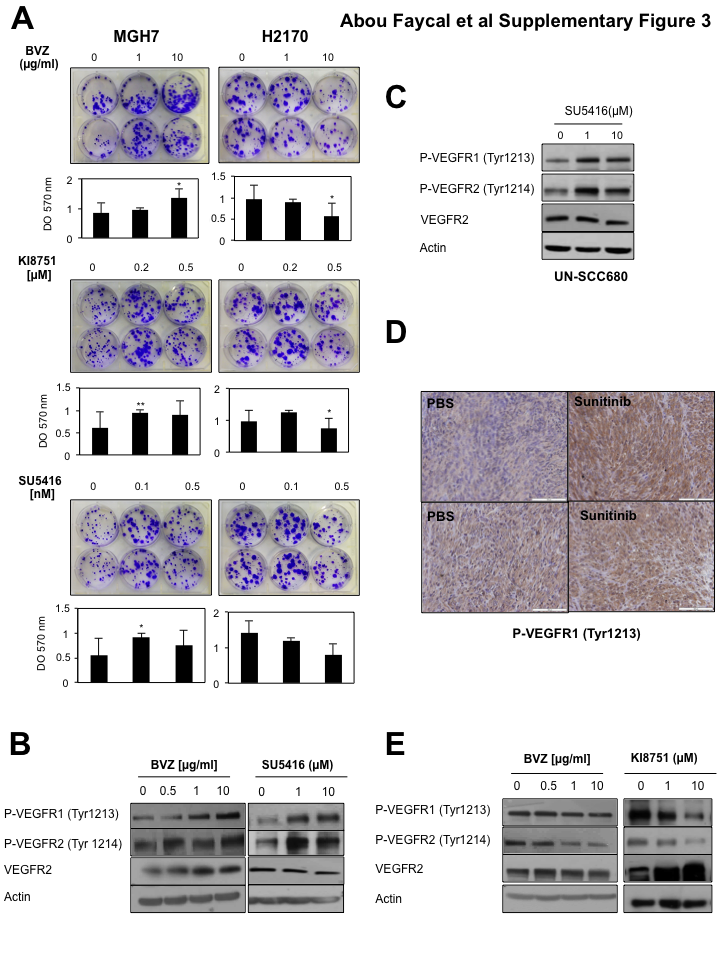

Supplement: Supplementary file 4 — Supplementary Figure 3 [file 41416_2018_128_MOESM4_ESM.tif]

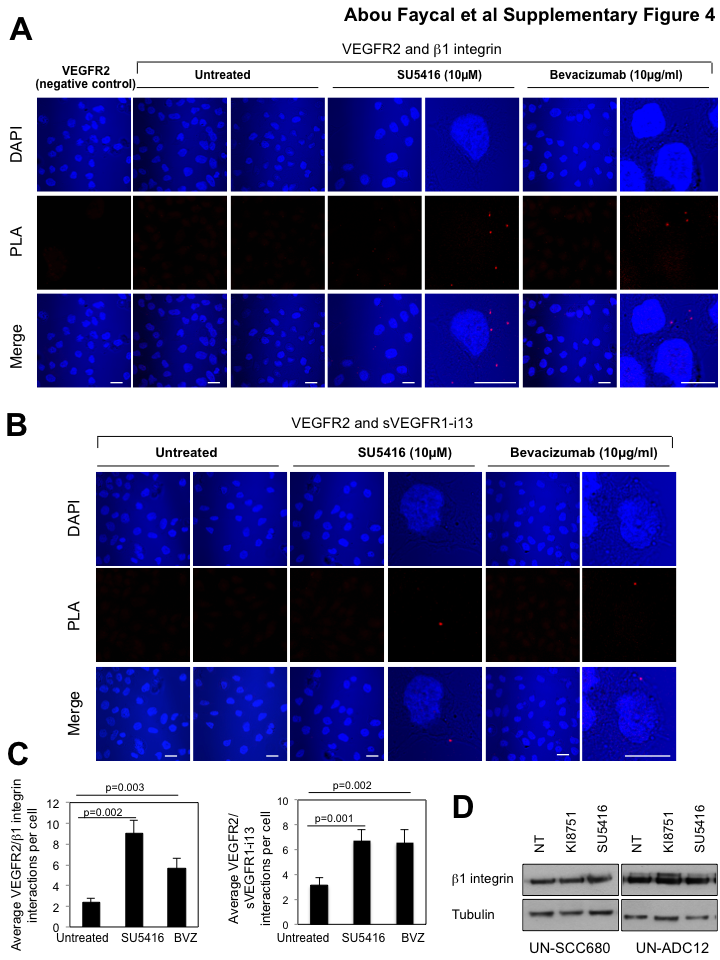

Supplement: Supplementary file 5 — Supplementary Figure 4 [file 41416_2018_128_MOESM5_ESM.tif]

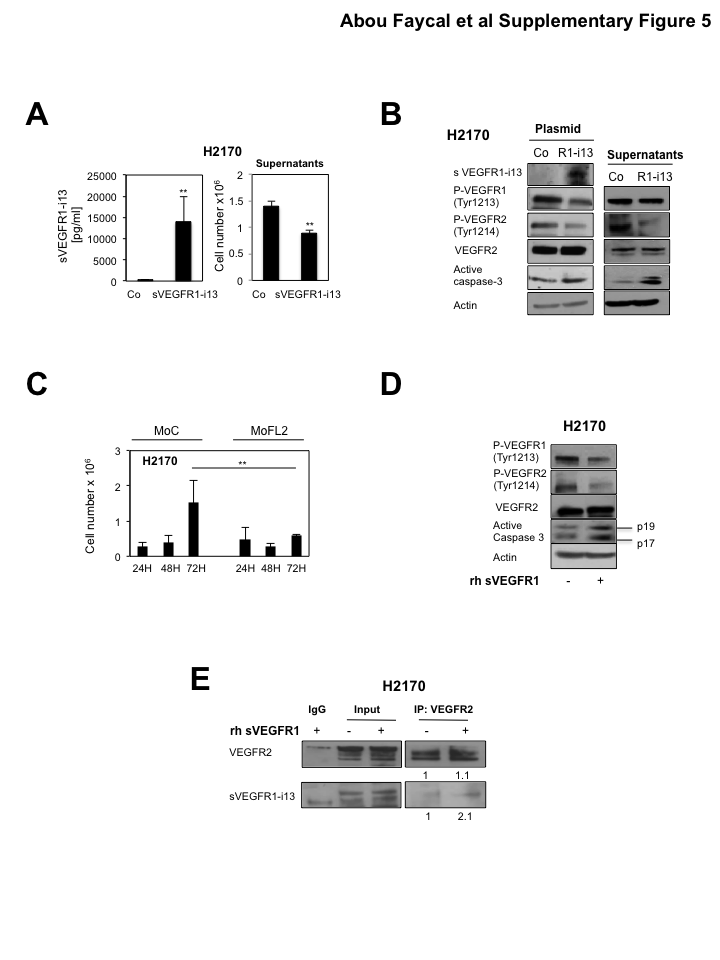

Supplement: Supplementary file 6 — Supplementary Figure 5 [file 41416_2018_128_MOESM6_ESM.tif]

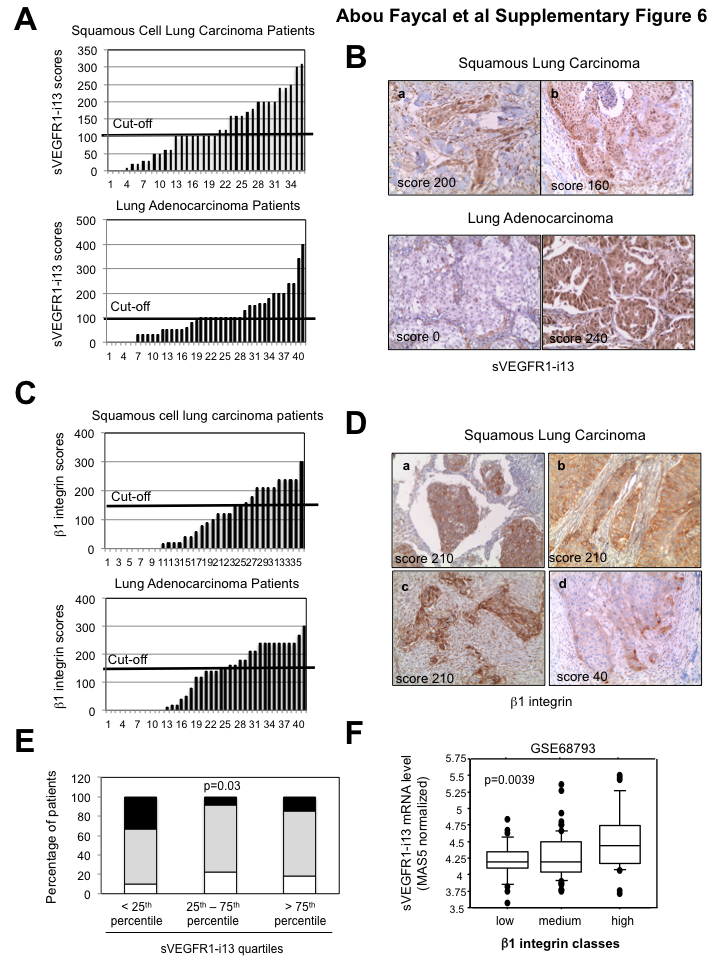

Supplement: Supplementary file 7 — Supplementary Figure 6 [file 41416_2018_128_MOESM7_ESM.tif]
